# Association of Adverse Childhood Experiences With Accelerated Epigenetic Aging in Midlife

**DOI:** 10.1001/jamanetworkopen.2023.17987

**Published:** 2023-06-12

**Authors:** Kyeezu Kim, Kristine Yaffe, David H. Rehkopf, Yinan Zheng, Drew R. Nannini, Amanda M. Perak, Jason M. Nagata, Greg E. Miller, Kai Zhang, Donald M. Lloyd-Jones, Brian T. Joyce, Lifang Hou

**Affiliations:** 1 Department of Preventive Medicine, Northwestern University Feinberg School of Medicine, Chicago, Illinois; 2 Department of Epidemiology and Biostatistics, University of California, San Francisco; 3 Department of Epidemiology and Population Health, Stanford University, Palo Alto, California; 4 Ann and Robert H. Lurie Children’s Hospital of Chicago, Chicago, Illinois; 5 Department of Pediatrics, University of California, San Francisco; 6 Department of Psychology, Northwestern University, Evanston, Illinois; 7 Department of Environmental Health Sciences, School of Public Health, University at Albany, State University of New York, Rensselaer; 8 Potocsnak Longevity Institute, Northwestern University Feinberg School of Medicine, Chicago, Illinois

## Abstract

**Importance:**

Adverse childhood experiences (ACEs) are associated with the risk of poorer health, and identifying molecular mechanisms may lay the foundation for health promotion in people with ACEs.

**Objective:**

To investigate the associations of ACEs with changes in epigenetic age acceleration (EAA), a biomarker associated with various health outcomes in middle-aged adults, in a population with balanced race and sex demographics.

**Design, Setting, and Participants:**

Data for this cohort study were from the Coronary Artery Risk Development in Young Adults (CARDIA) study. Participants in CARDIA underwent 8 follow-up exams from baseline (year 0 [Y0]; 1985-1986) to Y30 (2015-2016), and participant blood DNA methylation information was obtained at Y15 (2000-2001) and Y20 (2005-2006). Individuals from Y15 and Y20 with available DNA methylation data and complete variables for ACEs and covariates were included. Data were analyzed from September 2021 to August 2022.

**Exposures:**

Participant ACEs (general negligence, emotional negligence, physical violence, physical negligence, household substance abuse, verbal and emotional abuse, and household dysfunction) were obtained at Y15.

**Main Outcomes and Measures:**

The primary outcome consisted of results from 5 DNA methylation–based EAA measurements known to be associated with biological aging and long-term health: intrinsic EAA (IEAA), extrinsic EAA (EEAA), PhenoAge acceleration (PhenoAA), GrimAge acceleration (GrimAA), and Dunedin Pace of Aging Calculated From the Epigenome (DunedinPACE), measured at Y15 and Y20. Linear regression and generalized estimating equations were used to assess associations of the burden of ACEs (≥4 vs <4 ACEs) with EAA adjusting for demographics, health-related behaviors, and early life and adult socioeconomic status.

**Results:**

A total of 895 participants for Y15 (mean [SD] age, 40.4 [3.5] years; 450 males [50.3%] and 445 females [49.7%]; 319 Black [35.6%] and 576 White [64.4%]) and 867 participants for Y20 (mean [SD] age, 45.4 [3.5] years; 432 males [49.8%] and 435 females [50.2%]; 306 Black [35.3%] and 561 White [64.7%]) were included after excluding participants with missing data. There were 185 participants with (20.7%) vs 710 participants without (79.3%) 4 or more ACEs at Y15 and 179 participants with (20.6%) vs 688 participants without (79.4%) 4 or more ACEs at Y20. Having 4 or more ACEs was positively associated with EAA in years at Y15 (EEAA: β = 0.60 years; 95% CI, 0.18-1.02 years; PhenoAA: β = 0.62 years; 95% CI = 0.13-1.11 years; GrimAA: β = 0.71 years; 95% CI, 0.42-1.00 years; DunedinPACE: β = 0.01; 95% CI, 0.01-0.02) and Y20 (IEAA: β = 0.41 years; 95% CI, 0.05-0.77 years; EEAA: β = 1.05 years; 95% CI, 0.66-1.44 years; PhenoAA: β = 0.57 years; 95% CI, 0.08-1.05 years; GrimAA: β = 0.57 years; 95% CI, 0.28-0.87 years; DunedinPACE: β = 0.01; 95% CI, 0.01-0.02) after adjusting for demographics, health-related behaviors, and socioeconomic status.

**Conclusions and Relevance:**

In this cohort study, ACEs were associated with EAA among middle-aged adults after controlling for demographics, behavior, and socioeconomic status. These findings of the associations between early life experience and the biological aging process in midlife may contribute to health promotion in a life course perspective.

## Key Points

**Question:**

Do individuals with adverse childhood experiences (ACEs) have accelerated epigenetic age in their midlife?

**Findings:**

In this cohort study of 895 adults at year 15 and 867 adults at year 20 of follow-up with DNA methylation profiling, participants with 4 or more ACEs were likely to have older epigenetic ages compared with their chronological ages independently of their socioeconomic status in early or later life. ACEs was consistently associated with various epigenetic age acceleration measurements.

**Meaning:**

This study’s findings of consistent associations between a high burden of ACEs and multiple epigenetic age acceleration measurements suggest that ACEs may be persistently associated with the biological aging process in later life.

## Teaser

This cohort study investigates the association of adverse childhood experiences with 5 DNA methylation–based measures of accelerated epigenetic aging at 15 to 20 years of follow-up.

## Introduction

Adverse childhood experiences (ACEs) have been associated with a range of social disadvantages in adulthood.^1,2^ Individuals with a high burden of ACEs are more likely to engage in risky health behaviors,^3^ which can be associated with various age-related health outcomes.^3,4,5,6,7,8,9^ In a life-course perspective, ACEs like trauma in childhood were also associated with accelerated phenotypic and functional aging processes in later life.^10^ Detrimental outcomes associated with ACEs accumulate in a dose-response fashion, and experiencing 4 or more ACEs has been associated with various negative health outcomes.^8,11,12,13,14^ Studies on the molecular mechanisms of ACEs have found methylation changes in genes, including *NR3C1* and *FKBP5*, that are associated with glucocorticoid-mediated stress response,^15,16,17,18^ as well as genes, such as *SCL6A3*, *SKA2*, and *BDNF*, associated with other stress mediators.^19,20,21^ However, associations were limited to certain demographic subgroups in most studies, and underlying mechanisms remain elusive.^22^

Evidence has suggested that ACEs may modulate epigenetic pathways associated with biological aging and subsequently health-related outcomes. Prior studies observed associations between ACEs and shorter telomere length.^23,24,25,26^ More recently, researchers identified associations between ACEs and DNA methylation–based epigenetic age acceleration (EAA).^27,28,29,30,31,32,33^ DNA methylation reflects exposures throughout an individual’s life^34,35^; thus, EAA may serve as a useful tool to investigate associations between early life adversity and health in later life. Studies have shown associations of ACEs with EAA among youths.^27,29,30,31^ Researchers also have suggested that ACEs persisted and were associated with EAA in later life. A study^28^ found that childhood poverty and sexual abuse were associated with EAA in adults aged 50 years or older. Another study^32^ found positive associations among older females between having 4 or more ACEs and increased EAA. In Joshi et al,^33^ a higher burden of ACEs was associated with increased EAA among people at middle age or older.

Despite findings of prior research, few studies have examined the association between ACEs and EAA by race or sex, especially among adults.^28,32,33^ Furthermore, given that prior studies assessed the association of ACEs with EAA measured once, understanding the association over time remains challenging. To address these gaps, we investigated associations of ACEs with EAA using data on repeated measures of DNA methylation from the Coronary Artery Risk Development in Young Adults (CARDIA) study, which included subgroups with a balanced representation by race and sex.

## Methods

Written, informed consent was provided by CARDIA study participants. The CARDIA study protocol has been approved by the institutional review board (IRB) at each field center institution. Consent and IRB approval for the original CARDIA study extend to this cohort study. This study followed the Strengthening the Reporting of Observational Studies in Epidemiology (STROBE) reporting guideline).

### Data Source and Study Participants

The CARDIA study was a multicenter prospective cohort study across 4 field centers in the United States (Birmingham, Alabama; Chicago, Illinois; Minneapolis, Minnesota; and Oakland, California). At baseline (exam year 0 [Y0]; 1985-1986), 5115 male and female participants aged 18 to 30 years with self-reported Black or White race were enrolled, and 8 follow-ups were conducted until 2015 to 2016 (Y30). In the CARDIA study, Black and White participants were included to assess potential racial disparities in risk factors and outcomes of cardiovascular diseases. The CARDIA study included only Black and White individuals at the baseline and followed up those individuals; there was no exclusion of other races in this cohort study. The analytic data set of this study consisted of a subset of individuals from Y15 and Y20, when DNA methylation profiling was conducted. We included participants with available DNA methylation data and complete variables for ACEs and covariates (eFigure 1 in Supplement 1). Details of the CARDIA study and DNA methylation profiling are described in the eMethods in Supplement 1.

### Epigenetic Age Acceleration Estimation

This study included 5 epigenetic age estimators: intrinsic EAA (IEAA), extrinsic EAA (EEAA), PhenoAge acceleration (PhenoAA), GrimAge acceleration (GrimAA), and Dunedin Pace of Aging Calculated From the Epigenome (DunedinPACE). The first generation of epigenetic age estimators, intrinsic and extrinsic epigenetic age, were proposed as tools to estimate biological age. They captured cell-intrinsic properties of the aging process (intrinsic epigenetic age)^36^ and age-related changes in leukocyte composition (extrinsic epigenetic age).^37^ Second-generation estimators, PhenoAge and GrimAge, showed improved estimation of health and life span.^38,39^ EAA for these 4 measures was calculated from residuals derived from regressing each epigenetic age measure on chronological age. EAA values greater than 0 indicate that a participant’s epigenetic age is older than the individual’s chronological age (ie, the individual has accelerated epigenetic age). Lastly, DunedinPACE, a third-generation estimator, was developed to measure the pace of aging and represent the cross-sectional, ongoing rate of decline in physiology.^40^ The value of DunedinPACE is qualitatively different from the other measures, and values greater than 1 represent accelerated epigenetic age.

### Assessment of Adverse Childhood Experiences

In CARDIA, ACEs information was obtained at Y15 using the self-reported *Childhood Family Environment* questionnaire, based on the *Adverse Childhood Experiences* questionnaire developed by Felitti et al.^41^ The questionnaire was composed of 7 individual items asking how often a participant experienced each item prior to age 18 years: (1) the family knew what the participant was up to (general negligence), (2) felt loved (emotional negligence), (3) marked from getting hit (physical violence), (4) physical affection (physical negligence), (5) lived with an alcohol or drug abuser (household substance use problems), (6) sworn at or insulted (verbal or emotional abuse), and (7) well-organized house (household dysfunction). A Likert scale from 1 (rarely or none of the time) to 4 (most or all of the time) was used for each item in the original questionnaire,^42^ and items for general negligence, emotional negligence, physical negligence, and household dysfunction were reversely scored to give higher values for riskier family environments.

We dichotomized each ACE item based on a previous study^8^; any level of physical violence, verbal or emotional abuse, or household substance use problems was considered a risky environment. We dichotomized remaining items at the median to classify a risky environment (see eTable 1 in Supplement 1 for the distribution). Using dichotomized items, we further constructed a summary variable (≥4 ACEs vs <4 ACEs). We chose this threshold because prior research has reported increased risk of a variety of adverse health outcomes with 4 or more ACEs^8,11,12,13^ and this has become a widely accepted threshold.^14^ The distributions of mean EAA by number of ACEs in our data are shown in eFigure 2 in Supplement 1. The Cronbach α for the 4-level Likert scale and dichotomized variables was 0.76 and 0.71, respectively.

### Covariates

We included multiple sets of covariates in this study: basic demographics (chronological age, sex, race, and study center), early life socioeconomic status (SES; paternal occupation), health-related behaviors (smoking status, alcohol consumption, and physical activity), body mass index (BMI; calculated as weight in kilograms divided by height in meters squared), and SES in adulthood (education, marital status, and income). Among early life SES variables (paternal and maternal occupation and education), paternal occupation was included in downstream analyses given that it showed more than 10% changes in outcome estimates in fully adjusted models. More detailed information is in the eMethods in Supplement 1.

### Statistical Analysis

We performed descriptive analyses for participant characteristics at Y15 and Y20 by ACEs. We also explored pairwise Spearman correlations for the 7 individual ACE items.

To investigate associations between ACEs and EAA for Y15 to Y20, we modeled ACEs (≥4 vs <4 ACEs) as an independent variable and each EAA measure using a generalized estimating equations (GEE) model; we also examined each EAA measure at Y15 and Y20 as a separate dependent variable using a linear regression model. We adopted sequential models for both approaches. Point estimates (β) represent mean differences in EAA comparing participants with 4 or more vs less than 4 ACEs. Model 1 included basic demographics. Model 2 included early life SES in addition to covariates in model 1. Model 3 included participant health-related behaviors, BMI, and SES in adulthood, in addition to covariates in model 1. Finally, model 4 included all covariates in other models. Additionally, we investigated individual associations between the 7 dichotomized ACE items and 5 EAAs using model 4. We also assessed associations between ACEs and 5-year differences in epigenetic age using GEE models. The 5-year differences were calculated by subtracting epigenetic age at Y15 from Y20. In addition, we calculated rates of change by dividing 5-year epigenetic age differences by participant age differences in Y15 vs Y20. In GEE models, we incorporated the following covariates as time-dependent variables: age, BMI, smoking status, alcohol consumption, physical activity, education, marital status, and household income.

We performed stratified analyses to investigate whether the association between ACEs and EAA differed by race and sex. We examined the statistical significance of interactions between ACEs and participant race and sex by adding product terms in models without stratification. Finally, we performed multiple sensitivity analyses. We examined associations of ACEs with EAAs controlling additionally for estimated leukocyte proportions for second- and third generation estimators.^43^ Additionally, we adopted other forms of ACE variables: continuous ACEs (the summed score of 7 ACE items) and any ACEs (≥1 vs no ACEs).

Outcomes with 2-sided *P* value < .05 were considered statistically significant, and we further adopted Bonferroni correction (*P* value < .01 considered statistically significant, or .05 divided by 5, the number of EAA measures in the study) to address multiplicity potentially induced by multiple EAAs. Analyses were performed using SAS statistical software version 9.4 (SAS Institute). Data were analyzed from September 1, 2021, to August 31, 2022.

## Results

A total of 895 participants for Y15 (mean [SD] age, 40.4 [3.5] years; 450 males [50.3%] and 445 females [49.7%]; 319 Black [35.6%] and 576 White [64.4%]) and 867 participants for Y20 (mean [SD] age, 45.4 [3.5] years; 432 males [49.8%] and 435 females [50.2%]; 306 Black [35.3%] and 561 White [64.7%]) were included after excluding participants with missing data on EAA, ACEs, and other covariates. Table 1 presents participant characteristics by number of ACEs. There were 185 participants with (20.7%) vs 710 participants without (79.3%) 4 or more ACEs at Y15 and 179 participants with (20.6%) vs 688 participants without (79.4%) 4 or more ACEs at Y20. Pairwise correlations among 7 individual ACE items are displayed in eFigure 3 in Supplement 1.

**Table 1.** Characteristics of Study Participants

Characteristic	Participants, No. (%)			
	Year 15 (N = 895)		Year 20 (N = 867)	
	≥4 ACEs (n = 185)	<4 ACEs (n = 710)	≥4 ACEs (n = 179)	<4 ACEs (n = 688)
Age, mean (SD), y	40.3 (3.5)	40.4 (3.5)	45.5 (3.4)	45.4 (3.5)
Sex				
Males	91 (49.2)	359 (50.6)	87 (48.6)	345 (50.2)
Females	94 (50.8)	351 (49.4)	92 (51.4)	343 (49.8)
Race				
Black	68 (36.8)	251 (35.4)	66 (36.9)	240 (34.9)
White	117 (63.2)	459 (64.6)	113 (63.1)	448 (65.1)
Education, mean (SD), y	14.6 (2.4)	15.5 (2.5)	14.8 (2.3)	15.5 (2.5)
BMI, mean (SD)	28.3 (5.7)	28.1 (5.8)	24.3 (4.3)	24.1 (4.2)
Smoking status				
Never smoked	97 (52.4)	470 (62.2)	92 (51.4)	442 (64.2)
Former smoker	36 (19.5)	129 (18.2)	28 (15.6)	98 (14.3)
Current smoker	52 (28.1)	111 (15.6)	59 (33.0)	148 (21.5)
Alcohol consumption, mean (SD), mL/d	13.7 (22.5)	11.6 (22.1)	12.5 (24.3)	12.1 (20.1)
Physical activity total intensity score, mean (SD)	332.2 (263.2)	369.2 (286.3)	404.9 (280.8)	444 (294.6)
Father’s occupation				
Farmer or labor	34 (18.3)	123 (17.3)	33 (18.4)	119 (17.3)
Clerical, sales, stay-at-home parent	41 (22.2)	128 (18.0)	39 (21.8)	121 (17.6)
Executive or professional	85 (46.0)	377 (53.1)	83 (46.4)	370 (53.8)
Other	25 (13.5)	82 (11.6)	24 (13.4)	78 (11.3)
Married				
Yes	89 (48.1)	410 (57.7)	41 (22.9)	173 (25.2)
No	96 (51.9)	300 (42.3)	138 (77.1)	515 (74.8)
Annual household income, $				
<40 000	57 (30.8)	114 (16.1)	43 (24.0)	109 (15.8)
40 000-75 000	63 (34.1)	263 (37.0)	52 (29.1)	192 (27.9)
>75 000	65 (35.1)	333 (46.9)	84 (46.9)	387 (56.3)
ACE score, mean (SD)	17.3 (2.9)	9.8 (2.3)	17.3 (2.9)	9.8 (2.3)
ACEs, No.				
0	0	269 (37.9)	0	259 (37.7)
1	0	187 (26.3)	0	180 (26.1)
2	0	150 (21.1)	0	149 (21.7)
3	0	104 (14.7)	0	100 (14.5)
≥4	185 (100)	0	179 (100)	0
Study center				
Birmingham, AL	32 (17.3)	176 (24.8)	31 (17.3)	169 (24.6)
Chicago, IL	37 (20.0)	146 (20.6)	36 (20.1)	141 (20.5)
Minneapolis, MN	60 (32.4)	190 (26.7)	56 (31.3)	182 (26.4)
Oakland, CA	56 (30.3)	198 (27.9)	56 (31.3)	196 (28.5)

Abbreviations: BMI, body mass index (calculated as weight in kilograms divided by height in meters squared); ACE, adverse childhood experience.

Table 2 displays associations between ACEs and EAA over Y15 to Y20 using GEE models. Across models, we observed generally consistent magnitudes of EAA outcomes with IEAA, EEAA, and DunedinPACE (eg, for IEAA, β values were 0.25 years; 95% CI, −0.37 to 0.87 years for model 1; 0.17 years; 95% CI, −0.49 to 0.84 years for model 2; 0.21 years; 95% CI, −0.44 to 0.87 years for model 3; and 0.11 years; 95% CI, −0.59 to 0.82 years for model 4). Associations were found with PhenoAA in models with fewer covariates but not in models with additional covariates (eg, β values were 0.97 years; 95% CI, 0.05 to 1.87 years for model 1 but 0.53 years; 95% CI, −0.42 to 1.48 years for model 4). In the fully adjusted model (model 4), having 4 or more ACEs was associated with a mean increase in epigenetic age of 0.61 years in GrimAA (β = 0.61 years; 95% CI, 0.01 to 1.21 years) and 1% higher in DunedinPACE (β = 0.01; 95% CI, 0.01 to 0.03). No EAAs passed the Bonferroni correction threshold in model 4.

**Table 2.** Associations Between ACEs and EAA Over Years 15-20

EAA estimator	Model 1^a^		Model 2^a^		Model 3^a^		Model 4^a^	
	Mean EAA difference, β (95% CI)^b^	*P* value	Mean EAA difference, β (95% CI)^b^	*P* value	Mean EAA difference, β (95% CI)^b^	*P* value	Mean EAA difference, β (95% CI)^b^	*P* value
IEAA, y	0.25 (−0.37 to 0.87)	.43	0.17 (−0.49 to 0.84)	.60	0.21 (−0.44 to 0.87)	.52	0.11 (−0.59 to 0.82)	.75
EEAA, y	0.70 (−0.06 to 1.47)	.07	0.92 (0.11 to 1.72)	.03	0.57 (−0.20 to 1.36)	.15	0.74 (−0.08 to 1.56)	.08
PhenoAA, y	0.97 (0.05 to 1.87)	.03	0.83 (−0.11 to 1.78)	.08	0.74 (−0.16 to 1.65)	.11	0.53 (−0.42 to 1.48)	.27
GrimAA, y	1.32 (0.64 to 1.99)	<.001	1.52 (0.79 to 2.25)	<.001	0.50 (−0.05 to 1.06)	.08	0.61 (0.01 to 1.21)	.046
DunedinPACE	0.02 (0.01 to 0.04)	.002	0.03 (0.01 to 0.04)	.001	0.01 (−0.01 to 0.02)	.08	0.01 (0.01 to 0.03)	.04

Abbreviations: ACE, adverse childhood experience; DunedinPACE, Dunedin Pace of Aging Calculated From the Epigenome; EAA, epigenetic age acceleration; EEAA, extrinsic epigenetic age acceleration; GrimAA, GrimAge acceleration; IEAA, intrinsic epigenetic age acceleration; PhenoAA, PhenoAge acceleration.

^a^ Model 1 adjusted for basic demographics (age, sex, race, and study center). Model 2 adjusted for model 1 covariates and early life socioeconomic status (paternal occupation). Model 3 adjusted for model 1 covariates with health-related behaviors (smoking status, physical activity, and alcohol consumption) and body mass index. Model 4 adjusted for basic demographics, early life socioeconomic status, health-related behaviors and body mass index, and socioeconomic status in adulthood.

^b^ Point estimates (β) represent mean differences in EAA comparing participants with 4 or more vs less than 4 ACEs.

Table 3 shows associations for specific exam years between ACEs and EAA. For Y15, participants with 4 or more ACEs had increased EEAA (β = 0.60 years; 95% CI, 0.18-1.02 years), PhenoAA (β = 0.62 years; 95% CI, 0.13-1.11 years), GrimAA (β = 0.71 years; 95% CI, 0.42-1.00 years), and DunedinPACE (β = 0.01; 95% CI, 0.01-0.02) in model 4. We found similar associations for Y20 analysis (IEAA: β = 0.41 years; 95% CI, 0.05-0.77 years; EEAA: β = 1.05 years; 95% CI, 0.66-1.44 years; PhenoAA: β = 0.57 years; 95% CI, 0.08-1.05 years; GrimAA: β = 0.57 years; 95% CI, 0.28-0.87 years; DunedinPACE: β = 0.01; 95% CI, 0.01-0.02) in model 4. While we did not observe an association with IEAA at Y15, we found that 4 or more ACEs was associated with increased IEAA at Y20 (β = 0.41 years; 95% CI = 0.05-0.77 years) in model 4. Across models, robust associations were found between ACEs and DunedinPACE at Y15 (eg, β values for DunedinPACE were 0.03; 95% CI, 0.01-0.04 for model 1 and 0.01; 95% CI, 0.01-0.02 for model 4) and Y20 (eg, β values for DunedinPACE were 0.02; 95% CI, 0.01-0.04 in model 1 and 0.01; 95% CI, 0.01-0.02 in model 4). EEAA, GrimAA, and DunedinPACE passed the Bonferroni correction threshold in model 4 at Y15 (eg, EEAA: β = 0.60 years; 95% CI, 0.18-1.02 years; *P* = .004) and Y20 (eg, EEAA: β = 1.05 years; 95% CI, 0.66-1.44 years; *P* < .001). While there was less change in IEAA and DunedinPACE outcomes with adjustments for health-related behaviors and SES factors, other EAA measures generally showed attenuated magnitude of outcomes in models 2, 3, and 4.

**Table 3.** Associations Between ACEs and EAA at Year 15 and Year 20

EAA estimator	Model 1^a^		Model 2^a^		Model 3^a^		Model 4^a^	
	Mean EAA difference, β (95% CI)^b^	*P* value	Mean EAA difference, β (95% CI)^b^	*P* value	Mean EAA difference, β (95% CI)^b^	*P* value	Mean EAA difference, β (95% CI)^b^	*P* value
**Year 15**								
IEAA, y	0.04 (−0.69 to 0.78)	.91	0.05 (−0.72 to 0.84)	.89	−0.07 (−0.40 to 0.25)	.66	−0.07 (−0.42 to 0.28)	.70
EEAA, y	0.61 (−0.30 to 1.51)	.19	0.80 (−0.15 to 1.77)	.10	0.38 (−0.01 to 0.78)	.05	0.60 (0.18 to 1.02)	.004
PhenoAA, y	1.10 (0.06 to 2.13)	.04	0.95 (−0.16 to 2.07)	.10	0.75 (0.29 to 1.23)	.001	0.62 (0.13 to 1.11)	.01
GrimAA, y	1.49 (0.74 to 2.24)	<.001	1.71 (0.90-2.52)	<.001	0.52 (0.25 to 0.79)	<.001	0.71 (0.42 to 1.00)	<.001
DunedinPACE	0.03 (0.01 to 0.04)	.002	0.03 (0.01 to 0.05)	.001	0.01 (0.01 to 0.02)	.001	0.01 (0.01 to 0.02)	<.001
**Year 20**								
IEAA, y	0.53 (−0.30 to 1.36)	.21	0.38 (−0.51 to 1.28)	.40	0.56 (0.22 to 0.90)	.001	0.41 (0.05 to 0.77)	.02
EEAA, y	0.85 (−0.04 to 1.75)	.06	1.06 (0.10 to 2.02)	.03	0.81 (0.44 to 1.18)	<.001	1.05 (0.66 to 1.44)	<.001
PhenoAA, y	0.86 (−0.26 to 1.99)	.13	0.68 (−0.45 to 1.83)	.24	0.68 (0.22 to 1.15)	.003	0.57 (0.08 to 1.05)	.02
GrimAA, y	1.29 (0.47 to 2.11)	.002	1.46 (0.59 to 2.34)	.001	0.46 (0.18 to 0.74)	.001	0.57 (0.28 to 0.87)	<.001
DunedinPACE	0.02 (0.01 to 0.04)	.02	0.02 (0.01 to 0.05)	.01	0.01 (0.01 to 0.02)	.001	0.01 (0.01 to 0.02)	<.001

Abbreviations: ACEs, adverse childhood experiences; DunedinPACE, Dunedin Pace of Aging Calculated From the Epigenome; EAA, epigenetic age acceleration; EEAA, extrinsic epigenetic age acceleration; GrimAA, GrimAge acceleration; IEAA, intrinsic epigenetic age acceleration; PhenoAA, PhenoAge acceleration.

^a^ Model 1 adjusted for basic demographics (age, sex, race, and study center). Model 2 adjusted for model 1 covariates and early life socioeconomic status (paternal occupation). Model 3 adjusted for model 1 covariates with health-related behaviors (smoking status, physical activity, and alcohol consumption) and body mass index. Model 4 adjusted for basic demographics, early life socioeconomic status, health-related behaviors and body mass index, and socioeconomic status in adulthood.

^b^ Point estimates (β) represent mean differences in EAA comparing participants with 4 or more vs less than 4 ACEs.

Table 4 shows associations between ACEs and the rate of 5-year change in epigenetic age. In model 4, having 4 or more ACEs was associated with greater changes in first-generation estimators, intrinsic epigenetic age (β = 0.15 years; 95% CI, 0.06-0.24 years) and extrinsic epigenetic age (β = 0.14 years; 95% CI, 0.07-0.20 years), but not with second- or third-generation estimators. The Figure visualizes discrepancies between first-generation and second- and third-generation estimators in 5-year differences.

**Table 4.** Associations Between ACEs and Rate of 5-y Change in Epigenetic Age

EAA estimator	Model 1^a^		Model 2^a^		Model 3^a^		Model 4^a^	
	Mean EAA difference, β (95% CI)^b^	*P* value	Mean EAA difference, β (95% CI)^b^	*P* value	Mean EAA difference, β (95% CI)^b^	*P* value	Mean EAA difference, β (95% CI)^b^	*P* value
Intrinsic epigenetic age, y	0.14 (−0.10 to 0.39)	.27	0.13 (−0.13 to 0.40)	.33	0.16 (0.08 to 0.24)	<.001	0.15 (0.06 to 0.24)	<.001
Extrinsic epigenetic age, y	0.11 (−0.03 0.26)	.13	0.12 (−0.03 to 0.28)	.13	0.13 (0.06 to 0.19)	<.001	0.14 (0.07 to 0.20)	<.001
PhenoAge, y	−0.05 (−0.28 to 0.16)	.61	−0.03 (−0.27 to 0.20)	.77	−0.02 (−0.11 to 0.07)	.61	0.01 (−0.08 to 0.11)	.82
GrimAge, y	−0.02 (−0.17 to 0.11)	.71	−0.05 (−0.20 to 0.09)	.49	−0.01 (−0.06 to 0.03)	.58	−0.03 (−0.09 to 0.01)	.19
DunedinPACE	−0.01 (−0.01 to 0.01)	.48	−0.01 (−0.01 to 0.01)	.446	−0.01 (−0.01 to 0.00)	.331	−0.01 (−0.00 to 0.01)	.37

Abbreviations: ACEs, adverse childhood experiences; DunedinPACE, Dunedin Pace of Aging Calculated From the Epigenome; EAA, epigenetic age acceleration.

^a^ Model 1 adjusted for basic demographics (age, sex, race, and study center). Model 2 adjusted for model 1 covariates and early life socioeconomic status (paternal occupation). Model 3 adjusted for model 1 covariates with health-related behaviors (smoking status, physical activity, and alcohol consumption) and body mass index. Model 4 adjusted for basic demographics, early life socioeconomic status, health-related behaviors and body mass index, and socioeconomic status in adulthood.

^b^ Point estimates (β) represent mean differences in EAA comparing participants with 4 or ACEs vs less than 4 ACEs; 5-year change represents changes from year 15 to year 20.

**Figure.**
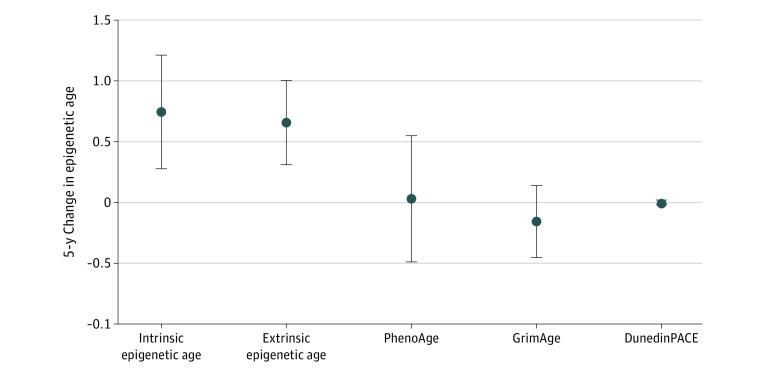
Associations Between Adverse Childhood Experiences and 5-Year Differences in Epigenetic Age Models were adjusted for age, sex, race, body mass index, smoking status, physical activity, alcohol consumption, education, marital status, income, paternal occupation, and study center. Point estimates (β) represent mean differences in epigenetic age comparing participants with 4 or more vs less than 4 adverse childhood experiences. DunedinPACE indicates Dunedin Pace of Aging Calculated From the Epigenome.

Results from subgroup analyses were qualitatively similar to our main results (eTables 2 and 3 in Supplement 1); however, PhenoAA for Y15 to Y20 showed an association among Black participants but not White participants (β = 2.22 years; 95% CI, 0.55 to 3.89 years vs β = −0.63 years; 95% CI, −1.78 to 0.50 years; *P* for interaction = .005). We found a similar pattern with PhenoAA at Y15.

Among 7 individual items for ACEs (eTables 4 and 5 in Supplement 1), emotional negligence was associated with increased EEAA over Y15 to Y20 (β = 0.84 years; 95% CI, 0.19-1.48 years). No individual items were specifically associated with other EAAs in GEE analysis or exam year–specific analysis. The sensitivity analysis additionally controlling for leukocyte composition was generally consistent with our main models (eTables 6 and 7 in Supplement 1). There were no associations with epigenetic age or there were associations with smaller changes in outcome in results from the continuous ACEs and any ACE analyses (eTables 8 and 9 in Supplement 1).

## Discussion

In this cohort study, we investigated associations of ACEs with EAA among middle-aged adults. Having 4 or more ACEs was associated with EAA at Y15 and Y20 after adjusting for demographics, health-related behaviors, and SES, suggesting that early life adversity was associated with lasting changes in biological aging processes. The associations of ACEs with IEAA, EEAA, and DunedinPACE were more robust across models. We observed changes in intrinsic and extrinsic epigenetic age from Y15 to Y20, suggesting the need for future research on early detection and intervention for later-life health outcomes associated with ACEs. Additionally, our findings suggest that future studies investigating the role of ACEs in life course-perspectives, including the contribution of ACEs to overall lifetime stress^44^ and the role of the resilience,^45^ and identifying psychosocial components associated with the greatest changes in EAA may aid in expanding the understanding of the association of ACEs with EAAs. Furthermore, adopting newer and developing EAA measurements (eg, GrimAge 2)^46^ in future studies may enhance insights gained. Finally, our results showed generally consistent associations across subgroups, suggesting that ACEs were associated with EAA outcomes regardless of demographics. However, we noted associations between ACEs and PhenoAA in Black participants but not White participants. The different proportion of race groups across EAA measurements may be 1 explanation.^36,37,38,39,40^ Future studies to develop EAA algorithms encompassing multiple races may help to better understand associations between ACEs and epigenetic modulations across race groups and contribute to positing an explanation for underlying biological or environmental factors that may be driving differences in our race-stratified analysis.

As prolonged and repetitive stress exposures in early life, ACEs may be associated with acceleration of the epigenetic aging processes via altered stress responses.^47,48^ Chronic exposures to stressful environments has been found to be associated with biological stability,^49^ dysregulation of the hypothalamic-pituitary-adrenal (HPA) axis, and dysfunctional physiological systems, including chronic inflammation, impaired cellular immunity, and disturbed glucose metabolism, which are also associated with epigenetic age estimators in this study.^50,51^ Intrinsic- and extrinsic epigenetic age estimators were designed to represent cell-intrinsic aging and immune system aging, respectively.^36,37^ Glucose is a component incorporated in PhenoAA, and leptin and growth differentiation factor 15, which are incorporated in GrimAA, play a role in glucose metabolism.^38,39^ Furthermore, many individual components that comprise PhenoAA, GrimAA, and DunedinPACE are known to be markers associated with inflammation.^38,39,40^ Taken together, these findings suggest that ACEs may be associated with increased EAA through pathways with disrupted cellular properties and immunity, inflammation, and endocrine system disruption owing to the exposure to toxic stress in childhood. This information may inform future interventions to reduce or mitigate deleterious epigenetic outcomes associated with ACEs.

Our findings were consistent with results from previous studies that observed associations of ACEs exposure with increased EAA,^27,28,29,30,31,32,33^ but our study provided an expanded view using multiple EAA measures with 2 time points. Repeated measurements of EAA and its changes over time in association with ACEs suggest that the role of ACEs may not have been static but rather dynamic over an individual’s lifetime. Furthermore, subtle variations between models with and without adjustment for health-related behaviors and SES in adulthood suggest that individuals with ACEs may have had an increased risk of accelerated biological aging, even with healthy behavior or social achievement in adulthood. We also found that ACEs were associated with multiple types of EAA measurements. Different EAA measures were developed with varying numbers of cytosine-phosphate-guanine (CpG) sites (IEAA: 353 CpGs; EEAA: 71 CpGs; PhenoAA: 513 CpGs; GrimAA: 1030 CpGs; and DunedinPACE: 173 CpGs) ^36,37,38,39,40^ and thus reflect different aspects of biological aging.^52^ Researchers have found that different EAAs were associated with different physiological characteristics.^52,53^ Our results with multiple EAA measures suggest that ACEs may play a role in various epigenetic pathways that may be associated with preclinical conditions and overt diseases. Altogether, our study results finding associations between early life experience and midlife EAAs that reflect various biological aging processes emphasize the need to monitor individuals with ACEs and provide interventions to prevent ACEs.

Consistent associations between ACEs and EAAs measured at 2 time points suggest that the association of ACEs with epigenetic dysregulation may persist later in life. Studies have found associations between ACEs and health outcomes in or after midlife,^4,5,6,7,8^ and our study findings suggest that epigenetic modification may play a role in the pathway. Additionally, ACEs were associated with 5-year changes in first-generation EAA measures, which incorporate epigenetic modulations associated with cell-intrinsic and immune system aging, but not with other measures, which incorporated more overt clinical outcomes. It is possible that cumulative stressors associated with ACEs may first take a part in epigenetic modulation related to cellular-level physiology, with subsequent disruption of phenotypic and clinical features. Coupled with the discrepancy between dynamic changes in first-generation estimators and static second- and third generation estimators over Y15 to Y20, these findings continue to suggest a direction for future studies with longitudinal methylation data covering wider stages of life. If differences in EAAs between examinations are smaller in earlier stages of participant lives than in midlife, that may suggest this time as an effective intervention point to prevent health outcomes associated with ACEs in later life. Identifying time windows in which ACEs-associated cumulative stressors are associated with epigenetic modulations may provide timelines for intervention to promote health in and after midlife among people with ACEs.

### Limitations

This study has several limitations. ACEs were measured at participant middle age by asking about experiences before age 18 years; thus, there is a possibility of recall bias. However, we believe that the bias would be toward the null given that our study outcome would not be expected to affect participant answers to the questionnaire. Second, ACEs measured in CARDIA did not include ACE elements of sexual abuse, parental separation or divorce, parental mental illness, or parental incarceration.^54^ These unaccounted elements may have induced residual confounding in associations found in our study. We additional note that residual confounding from other psychosocial components associated with ACEs, such as stress, resilience, and social network and support, may have affected our findings. Unmeasured ACEs and associated psychosocial components, such as resilience, may interact with each other and have synergetic or antagonistic associations with EAA. Future research assessing these and other unmeasured ACEs in association with EAA may expand our understanding of associations between ACEs and epigenetic aging.

## Conclusions

This cohort study found that ACEs were associated with accelerated epigenetic aging in later life controlling for early and later-life SES. Findings of associations of ACEs with multiple EAAs may have implications for potential association of ACEs with various aspects of biological aging. Future research to identify time frames between ACEs and EAA in broader time ranges in life may help clinicians build strategies for public health promotion and targeting populations in need.

## Notes

Supplement 1.
eMethods.

eReferences.
**eFigure 1.** Procedure to Select Eligible Study Participants **eFigure 2.** Mean Epigenetic Age Acceleration by Number of Adverse Childhood Experiences Items at Years 15 and 20 **eFigure 3.** Spearman Correlation in Childhood Family Environment Questionnaire **eTable 1.** Distributions of 7 Adverse Childhood Experiences Items Among Participants at Years 15 and 20 **eTable 2.** Associations Between Adverse Childhood Experiences and Epigenetic Age Acceleration Over Years 15-Y20 **eTable 3.** Associations Between Adverse Childhood Experiences and Epigenetic Age Acceleration at Years 15 and 20 **eTable 4.** Associations Between Dichotomized Individual Adverse Childhood Experiences Items and Epigenetic Age Acceleration Over Years 15-Y20 Using Generalized Estimating Equations Models **eTable 5.** Associations Between Dichotomized Individual Adverse Childhood Experiences Items and Epigenetic Age Acceleration at Years 15 and 20 **eTable 6.** Associations Between Adverse Childhood Experiences and Epigenetic Age Acceleration Over Years 15-20 Using Generalized Estimating Equations Models Additionally Adjusted for Leukocyte Composition **eTable 7.** Associations Between Adverse Childhood Experiences and Epigenetic Age Acceleration at Years 15 and 20 Additionally Adjusted for Leucocyte Composition **eTable 8.** Associations Between Continuous Adverse Childhood Experiences (Sum of Each Item Score) and Epigenetic Age Acceleration **eTable 9.** Associations Between Having any Adverse Childhood Experiences and Epigenetic Age Acceleration

Supplement 2.
Data Sharing Statement
