# Supplementary material for: Association of Adverse Childhood Experiences With Accelerated Epigenetic Aging in Midlife
Source: JAMA Netw Open. 2023 Jun 12;6(6):e2317987. doi: 10.1001/jamanetworkopen.2023.17987 (PMC10261996; doi:10.1001/jamanetworkopen.2023.17987)
Supplement: Supplement 1. — eMethods. eReferences. eFigure 1. Procedure to Select Eligible Study Participants eFigure 2. Mean Epigenetic Age Acceleration by Number of Adverse Childhood Experiences Items at Years 15 and 20 eFigure 3. Spearman Correlation in Childhood Family Environment Questionnaire eTable 1. Distributions of 7 Adverse Childhood Experiences Items Among Participants at Years 15 and 20 eTable 2. Associations Between Adverse Childhood Experiences and Epigenetic Age Acceleration Over Years 15-Y20 eTable 3. Associations Between Adverse Childhood Experiences and Epigenetic Age Acceleration at Years 15 and 20 eTable 4. Associations Between Dichotomized Individual Adverse Childhood Experiences Items and Epigenetic Age Acceleration Over Years 15-Y20 Using Generalized Estimating Equations Models eTable 5. Associations Between Dichotomized Individual Adverse Childhood Experiences Items and Epigenetic Age Acceleration at Years 15 and 20 eTable 6. Associations Between Adverse Childhood Experiences and Epigenetic Age Acceleration Over Years 15-20 Using Generalized Estimating Equations Models Additionally Adjusted for Leukocyte Composition eTable 7. Associations Between Adverse Childhood Experiences and Epigenetic Age Acceleration at Years 15 and 20 Additionally Adjusted for Leucocyte Composition eTable 8. Associations Between Continuous Adverse Childhood Experiences (Sum of Each Item Score) and Epigenetic Age Acceleration eTable 9. Associations Between Having any Adverse Childhood Experiences and Epigenetic Age Acceleration [file jamanetwopen-e2317987-s001.pdf]

Supplemental Online Content

Kim K, Yaffe K, Rehkopf DH, et al. Association of adverse childhood experiences with accelerated epigenetic aging in midlife. *JAMA Netw Open*. 2023;6(6):e2317987. doi:10.1001/jamanetworkopen.2023.17987

eMethods.

eReferences.

eFigure 1. Procedure to Select Eligible Study Participants

eFigure 2. Mean Epigenetic Age Acceleration by Number of Adverse Childhood Experiences Items at Years 15 and 20

eFigure 3. Spearman Correlation in Childhood Family Environment Questionnaire

eTable 1. Distributions of 7 Adverse Childhood Experiences Items Among Participants at Years 15 and 20

eTable 2. Associations Between Adverse Childhood Experiences and Epigenetic Age Acceleration Over Years 15-Y20

eTable 3. Associations Between Adverse Childhood Experiences and Epigenetic Age Acceleration at Years 15 and 20

eTable 4. Associations Between Dichotomized Individual Adverse Childhood Experiences Items and Epigenetic Age Acceleration Over Years 15-Y20 Using Generalized Estimating Equations Models

eTable 5. Associations Between Dichotomized Individual Adverse Childhood Experiences Items and Epigenetic Age Acceleration at Years 15 and 20

eTable 6. Associations Between Adverse Childhood Experiences and Epigenetic Age Acceleration Over Years 15-20 Using Generalized Estimating Equations Models Additionally Adjusted for Leukocyte Composition

eTable 7. Associations Between Adverse Childhood Experiences and Epigenetic Age Acceleration at Years 15 and 20 Additionally Adjusted for Leucocyte Composition

eTable 8. Associations Between Continuous Adverse Childhood Experiences (Sum of Each Item Score) and Epigenetic Age Acceleration

eTable 9. Associations Between Having any Adverse Childhood Experiences and Epigenetic Age Acceleration

This supplemental material has been provided by the authors to give readers additional information about their work.

© 2023 Kim K et al. *JAMA Network Open.*

Online Supplementary Materials: eMethods

The Coronary Artery Risk Development in Young Adults (CARDIA) Study

The CARDIA study is a population-based, multi-center prospective cohort study across four urban cities in the United States, initiated aiming to understand the factors (demographic, behavioral, and environmental) in association with the development of cardiovascular disease. The four field centers consist of Birmingham, AL, Chicago, IL, Minneapolis, MN, and Oakland, CA. The CARDIA study has been first launched in 1985-1986 (exam year [Y] 0), enrolling 5,115 men and women in age ranges of 18-30, with self-reported Black and White races. The CARDIA study protocol has been reviewed and approved by the Institutional Review Board of each study field center. The study design and recruitment of the CARDIA study have been described previously.[1] After baseline (Y0) exam, participants went through eight follow-up exams: 1987-1988 (Y2; N=4,624; mean age=26.9, SD=3.6), 1990-1991 (Y5; N=4,352; mean age=30.0, SD=3.6), 1992-1993 (Y7; N=4,086; mean age=32.0, SD=3.6), 1995-1996 (Y10; N=3,950; mean age=35.0, SD=3.7), 2000-2001 (Y15; N=3,672; mean age=40.2, SD=3.6), 2005-2006 (Y20; N=3,549; mean age=45.2, SD=3.6), 2010-2011 (Y25; N=3,499; mean age=50.2, SD=3.6), and 2015-2016 (Y30; N=3,358; mean age=55.1, SD=3.6).[2] The current study included participants with epigenetic age measurements from blood-based DNA methylation information obtained at Y15 and Y20.

DNA methylation profiling

In CARDIA, DNA methylation profiling using the Infinium Methylation EPIC BeadChip was performed in randomly selected 1,200 participants among 3,672 and 3,549 participants with available whole blood at both Y15 and Y20 exams. We performed quality control (QC) procedures by excluding CpGs with low detection rate (<95%; 6,209 CpGs were excluded), samples with low quality methylation measurements (>5% of CpGs) or low intensity of bisulfite conversion probe (<3 standard deviation [SD]; 87 samples were excluded), outliers using Tukey's method (<25th percentile – 3 * interquartile range (IQR) or >75th percentile + 3 * IQR; 95 samples were excluded).[3] R packages *minfi* [4] and *ENmix* [5] were used for QC and

© 2023 Kim K et al. *JAMA Network Open.*

preprocessing procedures for the DNA methylation data. As a result of these procedures, 1,999 samples from 1,042 participants were retained at Y15, and 957 at Y20, respectively, with 860,627 CpG probes in the final methylation dataset.

Covariates

We included the following covariates in the current study: chronological age, self-reported race and sex, study center, paternal occupation, smoking status, alcohol consumption, physical activity, body mass index (BMI), education, marital status, and annual household income. BMI was calculated from participants' anthropometric measurements of height and weight (kg/m2) at each exam. Information on other covariates was based on the questionnaire at each exam. For paternal occupation, participants were asked to answer the question, "What kind of work does (did) your father/the man responsible for you as a child do? What is (was) his job called?" and the verbatim answer has been recoded using Census Occupation Codes and classified into three groups: farmers or laborers, clerical, sales, housewife, and executive or professional. Smoking status was obtained from a self-reported questionnaire and then validated by serum cotinine levels, and classified into three categories: never, former, and current. Participants' daily alcohol consumption (mL/d) was calculated from their self-reported weekly consumption of beer, wine, and liquor. The total intensity score for physical activity was derived from the previous algorithm based on the frequency of activities with moderate and vigorous intensity in the past 12 months.[6] Participants were asked to report their maximum education years. Participants' marital status was obtained by asking whether they were married, widowed, divorced, separated, never been married, or living with someone in a marriage-like relationship, and recoded as yes (married) vs. no (all other categories) for the current study. Participants' self-reported annual household income was classified into three categories in this study: less than $40,000, $40,000-$75,000, and $75,000 or over.

eReferences

1. Friedman GD, Cutter GR, Donahue RP, et al. CARDIA: study design, recruitment, and some characteristics of the examined subjects. *J Clin Epidemiol* 1988; **41**(11): 1105-16.

© 2023 Kim K et al. *JAMA Network Open.*

2. Lloyd-Jones DM, Lewis CE, Schreiner PJ, Shikany JM, Sidney S, Reis JP. The Coronary Artery Risk Development In Young Adults (CARDIA) Study: JACC Focus Seminar 8/8. *J Am Coll Cardiol* 2021; **78**(3): 260-77.
3. J T. Exploratory Data Analysis: Pearson; 1997.
4. Aryee MJ, Jaffe AE, Corrada-Bravo H, et al. Minfi: a flexible and comprehensive Bioconductor package for the analysis of Infinium DNA methylation microarrays. *Bioinformatics (Oxford, England)* 2014; **30**(10): 1363-9.
5. Xu Z, Niu L, Li L, Taylor JA. ENmix: a novel background correction method for Illumina HumanMethylation450 BeadChip. *Nucleic Acids Res* 2016; **44**(3): e20.
6. Jacobs DR, Jr., Hahn LP, Haskell WL, Pirie P, Sidney S. Validity and Reliability of Short Physical Activity History: Cardia and the Minnesota Heart Health Program. *J Cardiopulm Rehabil* 1989; **9**(11): 448-59.

© 2023 Kim K et al. *JAMA Network Open.*

Supplementary Figure 1. Procedure to select eligible study participants.

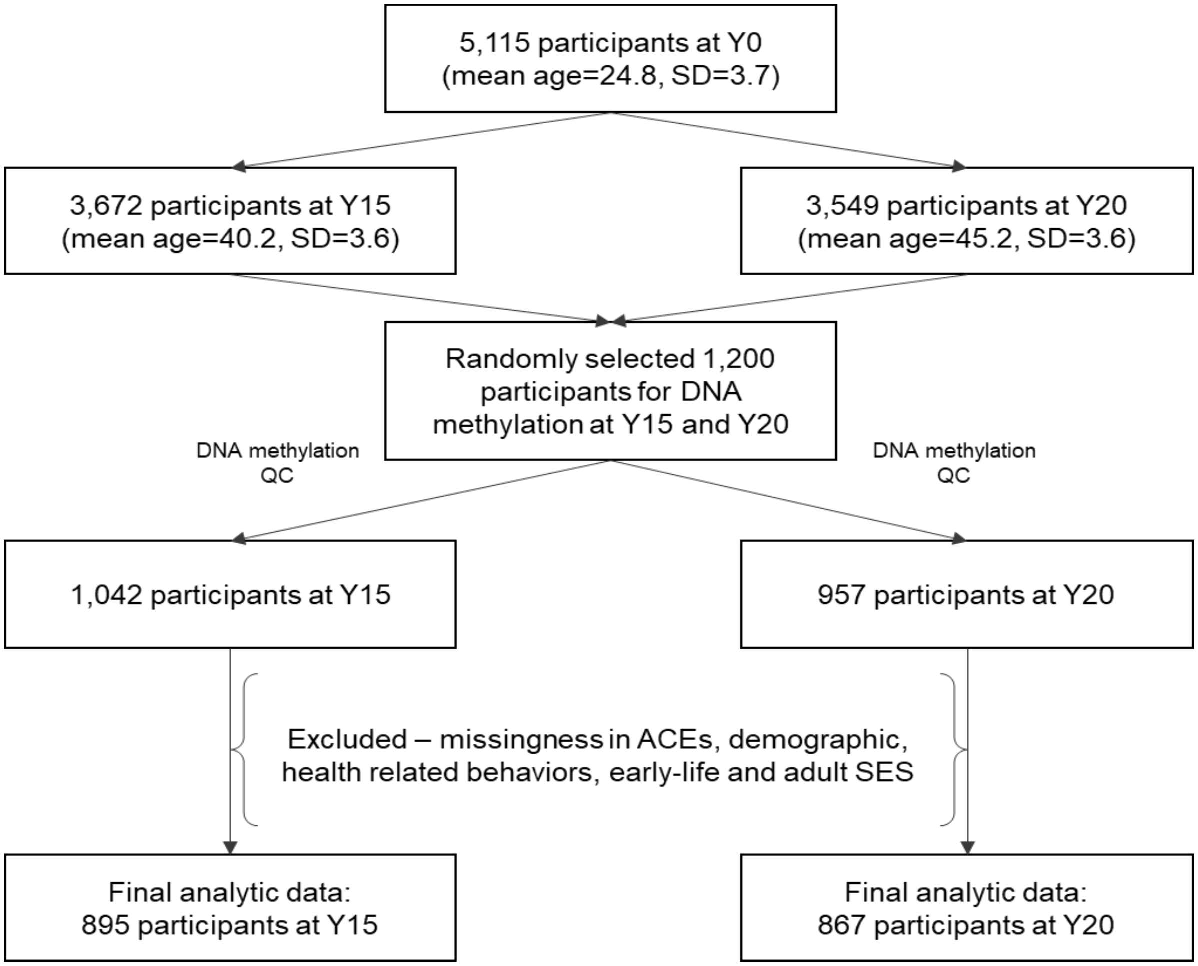

Note: SD: standard deviation; QC: quality control; ACEs: adverse childhood experiences; SES: socioeconomic status.

© 2023 Kim K et al. *JAMA Network Open.*

Supplementary Figure 2. Mean EAA by number of ACE items at Y15 (2-A) and Y20 (2-B).

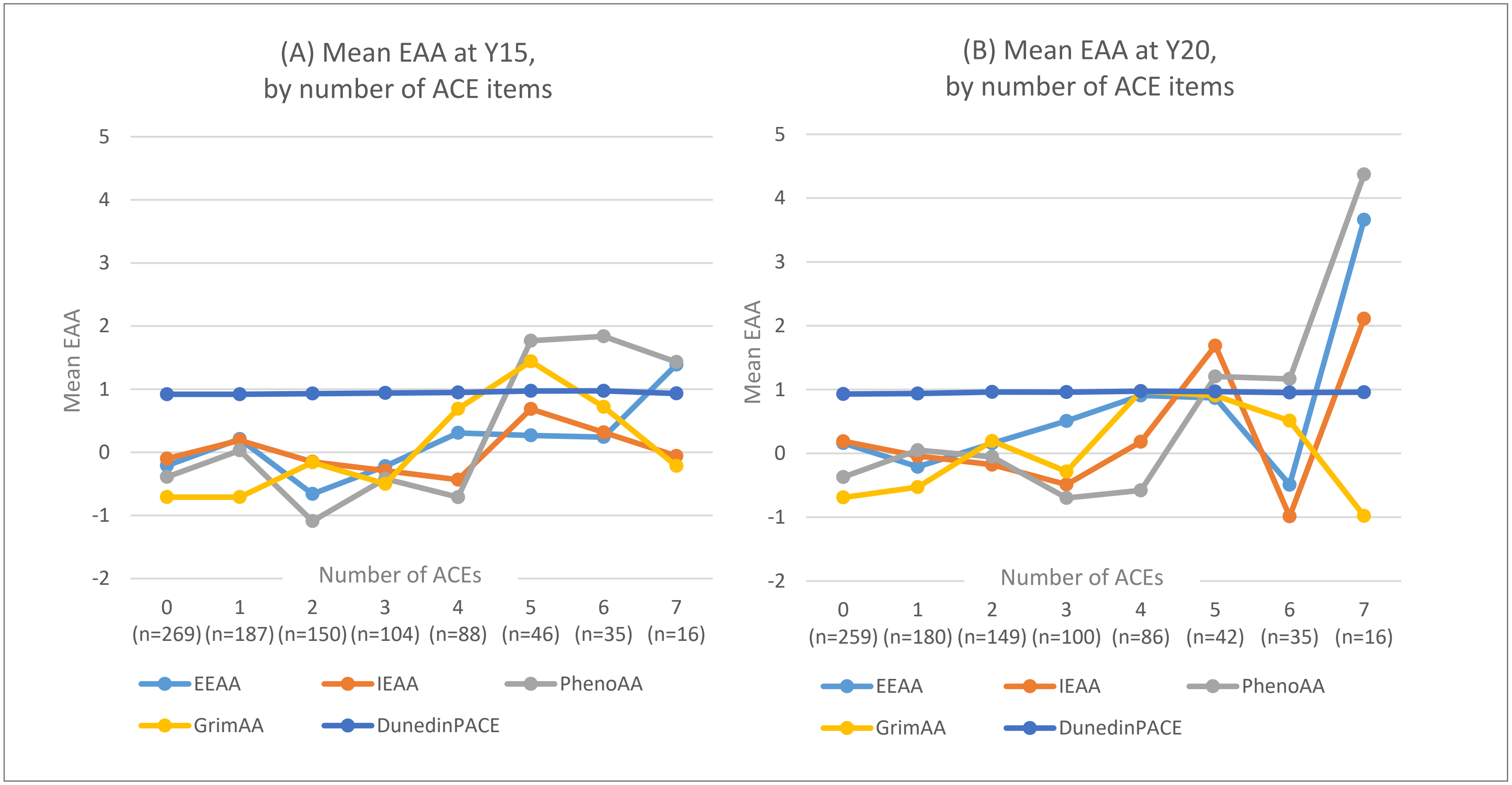

© 2023 Kim K et al. *JAMA Network Open.*

Supplementary Figure 3. Spearman's correlation among childhood family environment questionnaire.

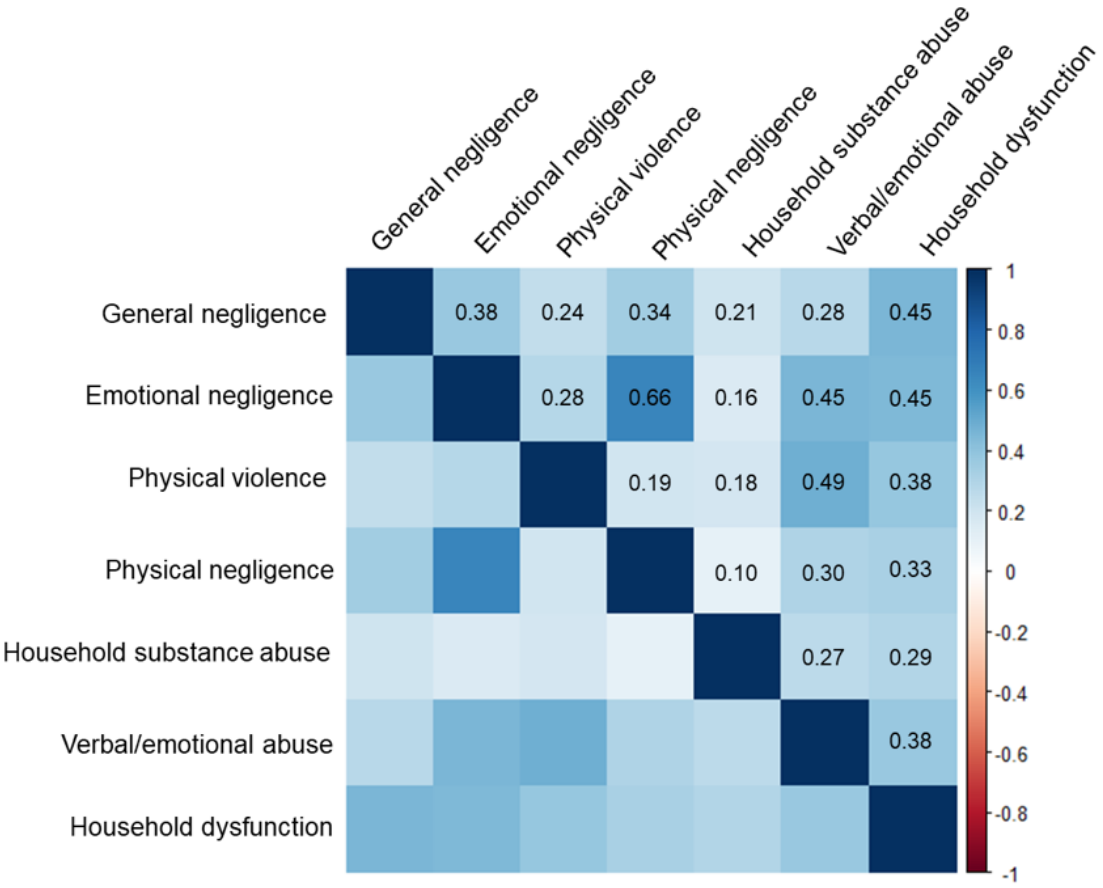

Note: P-values were <0.05 for all pairwise correlation coefficients.

© 2023 Kim K et al. *JAMA Network Open.*

Supplementary Table 1. Distributions of seven ACE items among CARDIA participants at Y15 and Y20

ACE item	Y15<br>N (%)	Y20<br>N (%)
General negligence (Did family know what you were up to?)		
Rarely or none of the time	46 (5.1)	45 (5.2)
Some or little of the time	145 (16.2)	141 (16.3)
Occasionally or moderate amount of time	270 (30.2)	259 (29.8)
Most or all of the time	434 (48.5)	422 (48.7)
Emotional negligence (How often did you feel loved?)		
Rarely or none of the time	19 (2.1)	19 (2.2)
Some or little of the time	92 (10.3)	87 (10.0)
Occasionally or moderate amount of time	217 (24.3)	212 (24.5)
Most or all of the time	567 (63.3)	549 (63.3)
Physical violence (How often marked from getting hit?)		
Rarely or none of the time	740 (82.7)	715 (82.5)
Some or little of the time	97 (10.8)	96 (11.1)
Occasionally or moderate amount of time	51 (5.7)	50 (5.8)
Most or all of the time	7 (0.8)	6 (0.7)
Physical negligence (How often did you get physical affection?)		
Rarely or none of the time	94 (10.5)	92 (10.6)

© 2023 Kim K et al. *JAMA Network Open.*

Some or little of the time	178 (19.9)	172 (19.8)
Occasionally or moderate amount of time	285 (31.8)	278 (32.1)
Most or all of the time	338 (37.8)	325 (37.5)
Household substance abuse (How often did you live with alcohol/drug abuser?)		
Rarely or none of the time	647 (72.3)	629 (72.5)
Some or little of the time	76 (8.5)	73 (8.4)
Occasionally or moderate amount of time	72 (8.0)	69 (8.0)
Most or all of the time	100 (11.2)	96 (11.1)
Verbal/emotional abuse (How often sworn or insulted?)		
Rarely or none of the time	543 (60.7)	526 (60.7)
Some or little of the time	183 (20.5)	178 (20.5)
Occasionally or moderate amount of time	139 (15.5)	135 (15.6)
Most or all of the time	30 (3.3)	28 (3.2)
Household dysfunction (Was your house well organized?)		
Rarely or none of the time	51 (5.7)	51 (5.9)
Some or little of the time	106 (11.8)	104 (12.0)
Occasionally or moderate amount of time	219 (24.5)	208 (24.0)
Most or all of the time	519 (58.0)	504 (58.1)

Note: Questions for general negligence, emotional negligence, physical negligence, and household dysfunction were recoded to have higher values for riskier family environment.

© 2023 Kim K et al. *JAMA Network Open.*

Supplementary Table 2. Associations between ACEs (having 4+ ACEs vs. <4 ACEs) and EAA over Y15-Y20 using GEE models, by race- and sex subgroups in CARDIA.

EAA estimators	By race					By sex				
	Black participants		White participants		*P*-interaction for race	Men		Women		*P*-interaction for sex
	β (95% CI)	*P*-value	*β* (95% CI)	*P*-value		*β* (95% CI)	*P*-value	*β* (95% CI)	*P*-value	
IEAA	0.82 (-0.61, 2.26)	0.261	-0.57 (-1.56, 0.41)	0.255	0.249	0.78 (-0.48, 2.05)	0.225	-0.85 (-1.91, 0.20)	0.114	0.378
EEAA	1.25 (-0.28, 2.78)	0.111	0.51 (-0.43, 1.47)	0.289	0.459	0.35 (-0.93, 1.64)	0.588	1.17 (0.10, 2.24)	0.031	0.321
PhenoAA	2.22 (0.55, 3.89)	0.009†	-0.63 (-1.78, 0.50)	0.276	0.005†	0.11 (-1.26, 1.50)	0.868	0.67 (-0.66, 2.02)	0.324	0.816
GrimAA	0.76 (-0.27, 1.81)	0.150	0.54 (-0.18, 1.28)	0.145	0.855	0.56 (-0.31, 1.44)	0.206	0.64 (-0.22, 1.50)	0.147	0.822
DunedinPACE	0.01 (-0.02, 0.03)	0.601	0.02 (0.00, 0.04)	0.041	0.345	0.01 (-0.01, 0.03)	0.236	0.01 (-0.01, 0.04)	0.128	0.894

Note: Models adjusted for age, sex, race, BMI, education, marital status, income, smoking status, physical activity, alcohol consumption, paternal occupation, and study center. Point estimates (β) represent differences in EAA comparing participants with 4+ ACEs vs. <4 ACEs. ACEs: adverse childhood experiences; SES: socioeconomic status. † *P*-value less than 0.01 (Bonferroni correction threshold).

© 2023 Kim K et al. *JAMA Network Open.*

Supplementary Table 3. Associations between ACEs (having 4+ ACEs vs. <4 ACEs) and EAA at Y15 and Y20, by race- and sex subgroups in CARDIA.

EAA estimators	By race					By sex				
	Black participants		White participants		*P*-interaction for race	Men		Women		*P*-interaction for sex
	β (95% CI)	*P*-value	β (95% CI)	*P*-value		β (95% CI)	*P*-value	β (95% CI)	*P*-value	
Epigenetic age acceleration at Y15										
IEAA	0.96 (-0.46, 2.38)	0.185	-0.52 (-1.45, 0.41)	0.273	0.064	0.77 (-0.43, 1.97)	0.209	-0.95 (-1.88, -0.02)	0.044	0.024
EEAA	1.45 (-0.39, 3.30)	0.122	0.12 (-0.88, 1.14)	0.802	0.214	0.37 (-1.09, 1.84)	0.619	0.29 (-0.85, 1.43)	0.616	0.812
PhenoAA	3.07 (1.36, 4.78)	<.001†	-0.87 (-2.22, 0.46)	0.201	<.001†	0.85 (-0.77, 2.47)	0.304	0.15 (-1.19, 1.49)	0.823	0.416
GrimAA	0.18 (-0.91, 1.27)	0.741	0.92 (0.12, 1.72)	0.023	0.263	0.58 (-0.47, 1.63)	0.279	0.72 (-0.04, 1.49)	0.063	0.466
DunedinPACE	-0.01 (-0.03, 0.02)	0.766	0.02 (0.01, 0.04)	0.013	0.051	0.01 (-0.01, 0.04)	0.364	0.01 (-0.01, 0.03)	0.318	0.933
Epigenetic age acceleration at Y20										
IEAA	0.64 (-0.94, 2.22)	0.428	0.16 (-0.91, 1.24)	0.761	0.683	-0.02 (-1.30, 1.25)	0.968	0.66 (-0.49, 1.81)	0.262	0.497
EEAA	1.46 (-0.27, 3.21)	0.098	0.93 (-0.24, 2.10)	0.119	0.686	0.23 (-1.20, 1.67)	0.747	1.74 (0.50, 2.98)	0.006	0.051
PhenoAA	1.82 (-0.31, 3.97)	0.094	-0.37 (-1.70, 0.95)	0.582	0.101	-0.84 (-2.49, 0.80)	0.316	1.42 (-0.03, 2.88)	0.055	0.169
GrimAA	1.09 (-0.29, 2.48)	0.121	0.21 (-0.58, 1.02)	0.594	0.299	0.11 (-0.88, 1.12)	0.820	0.82 (-0.17, 1.83)	0.106	0.557
DunedinPACE	0.02 (-0.01, 0.05)	0.206	0.01 (-0.01, 0.03)	0.284	0.749	0.01 (-0.01, 0.03)	0.398	0.01 (-0.01, 0.04)	0.173	0.994

Note: Models adjusted for age, sex, race, BMI, education, marital status, income, smoking status, physical activity, alcohol consumption, paternal occupation, and study center. Point estimates (β) represent differences in EAA comparing participants with 4+ ACEs vs. <4 ACEs. † *P*-value less than 0.01 (Bonferroni correction threshold).

© 2023 Kim K et al. *JAMA Network Open.*

Supplementary Table 4. Associations between dichotomized individual ACE items and EAA over Y15-Y20 using GEE models.

ACE item (Original question)	IEAA		EEAA		PhenoAA		GrimAA		DunedinPACE	
	β (95% CI)	*P*-value	β (95% CI)	*P*-value	β (95% CI)	*P*-value	β (95% CI)	*P*-value	β (95% CI)	*P*-value
General negligence *(Did family know what you were up to?)*	0.44 (-0.38, 1.27)	0.294	0.46 (-0.36, 1.28)	0.269	0.68 (-0.33, 1.69)	0.188	0.45 (-0.19, 1.10)	0.174	0.02 (0.01, 0.04)	0.009
Emotional negligence *(How often did you feel loved?)*	-0.13 (-0.80, 0.53)	0.699	0.84 (0.19, 1.48)	0.010†	0.49 (-0.27, 1.26)	0.206	0.37 (-0.10, 0.86)	0.125	0.01 (-0.01, 0.02)	0.252
Physical violence *(How often marked from getting hit?)*	0.22 (-0.59, 1.04)	0.587	0.54 (-0.33, 1.42)	0.225	0.23 (-0.71, 1.18)	0.624	-0.28 (-0.88, 0.31)	0.353	0.01 (-0.01, 0.02)	0.310
Physical negligence *(How often did you get physical affection?)*	-0.51 (-1.18, 0.15)	0.130	-0.06 (-0.73, 0.61)	0.860	0.31 (-0.48, 1.12)	0.438	0.48 (-0.02, 0.99)	0.063	0.02 (0.01, 0.03)	0.004
Household substance abuse *(How often did you live with alcohol/drug abuser?)*	0.01 (-0.66, 0.67)	0.989	-0.02 (-0.74, 0.68)	0.941	-0.04 (-0.86, 0.77)	0.917	0.05 (-0.48, 0.58)	0.852	0.01 (-0.01, 0.02)	0.390
Verbal/emotional abuse *(How often sworn or insulted?)*	-0.29 (-0.92, 0.34)	0.366	0.55 (-0.08, 1.19)	0.088	-0.02 (-0.78, 0.72)	0.944	0.05 (-0.40, 0.51)	0.822	0.01 (-0.01, 0.02)	0.188
Household dysfunction *(Was your house well organized?)*	-0.12 (-0.99, 0.74)	0.775	0.05 (-0.82, 0.94)	0.898	-0.20 (-1.27, 0.87)	0.713	-0.13 (-0.81, 0.54)	0.695	-0.01 (-0.03, 0.01)	0.062

Note: Models adjusted for age, sex, race, BMI, education, marital status, income, smoking status, physical activity, alcohol consumption, paternal occupation, and study center. Point estimates (β) represent differences in EAA comparing participants with risky vs. non-risky for each item. † *P*-value less than 0.01 (Bonferroni correction threshold).

© 2023 Kim K et al. *JAMA Network Open.*

Supplementary Table 5. Associations between dichotomized individual ACE items and EAA at Y15 and Y20.

ACE item (Original question)	IEAA		EEAA		PhenoAA		GrimAA		DunedinPACE	
	β (95% CI)	*P*-value	β (95% CI)	*P*-value	β (95% CI)	*P*-value	β (95% CI)	*P*-value	β (95% CI)	*P*-value
Associations between ACEs and Y15 EAA										
General negligence *(Did family know what you were up to?)*	0.37 (-0.44, 1.19)	0.369	0.58 (-0.09, 1.27)	0.326	1.05 (-0.09, 2.20)	0.071	0.58 (-0.09, 1.27)	0.091	0.01 (-0.01, 0.03)	0.110
Emotional negligence *(How often did you feel loved?)*	-0.18 (-0.82, 0.45)	0.563	0.45 (0.05, 0.97)	0.033	0.61 (-0.24, 1.46)	0.161	0.45 (-0.05, 0.97)	0.080	0.01 (-0.01, 0.02)	0.255
Physical violence *(How often marked from getting hit?)*	0.35 (-0.43, 1.14)	0.373	-0.14 (-0.78, 0.50)	0.547	0.07 (-1.04, 1.19)	0.897	-0.14 (-0.78, 0.50)	0.666	0.01 (-0.01, 0.02)	0.327
Physical negligence *(How often did you get physical affection?)*	-0.52 (-1.16, 0.11)	0.105	0.42 (-0.10, 0.94)	0.601	0.61 (-0.25, 1.49)	0.167	0.42 (-0.10, 0.94)	0.116	0.02 (0.01, 0.03)	0.007
Household substance abuse *(How often did you live with alcohol/drug abuser?)*	-0.02 (-0.68, 0.63)	0.942	0.10 (-0.46, 0.67)	0.382	-0.42 (-1.34, 0.50)	0.370	0.10 (-0.46, 0.67)	0.725	0.01 (-0.01, 0.02)	0.339
Verbal/emotional abuse *(How often sworn or insulted?)*	-0.28 (-0.90, 0.32)	0.362	0.16 (-0.33, 0.67)	0.234	0.15 (-0.70, 1.00)	0.730	0.16 (-0.33, 0.67)	0.515	0.01 (-0.01, 0.02)	0.251
Household dysfunction *(Was your house well organized?)*	-0.04 (-0.87, 0.78)	0.917	-0.14 (-0.83, 0.53)	0.845	-0.04 (-1.22, 1.13)	0.942	-0.14 (-0.83, 0.53)	0.670	-0.01 (-0.03, 0.01)	0.088
Associations between ACEs and Y20 EAA										
General negligence *(Did family know what you were up to?)*	0.41 (-0.47, 1.30)	0.365	0.49 (-0.47, 1.45)	0.318	0.35 (-0.87, 1.57)	0.573	0.45 (-0.33, 1.23)	0.257	0.02 (0.01, 0.04)	0.013
Emotional negligence *(How often did you feel loved?)*	0.37 (-0.35, 1.10)	0.317	0.93 (0.14, 1.72)	0.021	0.54 (-0.42, 1.50)	0.271	0.28 (-0.29, 0.85)	0.335	0.01 (-0.01, 0.02)	0.530
Physical violence *(How often marked from getting hit?)*	0.39 (-0.64, 1.43)	0.458	0.75 (-0.24, 1.76)	0.140	0.60 (-0.55, 1.76)	0.306	-0.14 (-0.83, 0.55)	0.686	0.01 (-0.01, 0.03)	0.177
Physical negligence *(How often did you get physical affection?)*	-0.35 (-1.16, 0.44)	0.386	0.41 (-0.39, 1.22)	0.318	0.31 (-0.68, 1.31)	0.534	0.42 (-0.19, 1.04)	0.180	0.02 (0.01, 0.03)	0.021
Household substance abuse *(How often did you live with alcohol/drug abuser?)*	0.46 (-0.30, 1.23)	0.233	0.41 (-0.45, 1.28)	0.349	0.27 (-0.71, 1.26)	0.585	0.07 (-0.54, 0.69)	0.808	0.01 (-0.01, 0.02)	0.489
Verbal/emotional abuse *(How often sworn or insulted?)*	-0.27 (-1.00, 0.44)	0.451	0.77 (-0.01, 1.55)	0.051	-0.07 (-1.02, 0.88)	0.883	0.01 (-0.53, 0.57)	0.943	0.01 (-0.01, 0.02)	0.254
Household dysfunction *(Was your house well organized?)*	-0.16 (-1.12, 0.78)	0.730	0.01 (-1.04, 1.04)	0.999	0.04 (-1.23, 1.32)	0.950	-0.16 (-1.01, 0.67)	0.694	-0.01 (-0.04, 0.01)	0.065

Note: Models adjusted for age, sex, race, BMI, education, marital status, income, smoking status, physical activity, alcohol consumption, paternal occupation, and study center. Point estimates (β) represent differences in EAA comparing participants with risky vs. non-risky for each item.

© 2023 Kim K et al. *JAMA Network Open.*

Supplementary Table 6. Associations between ACEs (having 4+ ACEs vs. <4 ACEs) and epigenetic age acceleration (EAA) over Y15-Y20 using generalized estimating equations (GEE) models, additionally adjusted for leukocyte composition.

EAA estimators	Adjusted for demographics, early life SES, health-related behaviors and BMI, adulthood SES, and leukocyte composition	
	β (95% CI)	*P*-value
PhenoAA	0.52 (-0.34, 1.39)	0.237
GrimAA	0.59 (0.01, 1.17)	0.047
DunedinPACE	0.01 (0.01, 0.03)	0.022

Note: Point estimates (β) represent differences in EAA comparing participants with 4+ ACEs vs. <4 ACEs. ACEs: adverse childhood experiences; SES: socioeconomic status. † *P*-value less than 0.01 (Bonferroni correction threshold).

© 2023 Kim K et al. *JAMA Network Open.*

Supplementary Table 7. Associations between ACEs (having 4+ ACEs vs. <4 ACEs) and epigenetic age acceleration (EAA) at Y15 and Y20, additionally adjusted for leukocyte composition.

EAA estimators	Adjusted for demographics, early life SES, health-related behaviors and BMI, adulthood SES, and leukocyte composition	
	β (95% CI)	*P*-value
Epigenetic age acceleration at Y15		
PhenoAA	0.62 (0.16, 1.08)	0.007†
GrimAA	0.71 (0.43, 0.99)	<.001†
DunedinPACE	0.01 (0.01, 0.02)	<.001†
Epigenetic age acceleration at Y20		
PhenoAA	0.52 (0.07, 0.97)	0.023
GrimAA	0.51 (0.22, 0.79)	<.001†
DunedinPACE	0.01 (0.01, 0.02)	<.001†

Note: Point estimates (β) represent differences in EAA comparing participants with 4+ ACEs vs. <4 ACEs. ACEs: adverse childhood experiences; SES: socioeconomic status. † *P*-value less than 0.01 (Bonferroni correction threshold).

© 2023 Kim K et al. *JAMA Network Open.*

Supplementary Table 8. Associations between continuous ACE (sum of each ACE item score) and epigenetic age acceleration (EAA) at Y15 and Y20

EAA estimators	Adjusted for demographics, early life SES, health-related behaviors and BMI, and adulthood SES	
	β (95% CI)	*P*-value
GEE models		
IEAA	0.01 (-0.05, 0.08)	0.743
EEAA	0.06 (-0.02, 0.15)	0.159
PhenoAA	0.03 (-0.06, 0.12)	0.532
GrimAA	0.01 (-0.04, 0.07)	0.600
DunedinPACE	0.01 (-0.01, 0.00)	0.218
Epigenetic age acceleration at Y15		
IEAA	0.01 (-0.02, 0.05)	0.475
EEAA	0.04 (-0.01, 0.08)	0.054
PhenoAA	0.06 (-0.01, 0.10)	0.690
GrimAA	0.02 (-0.01, 0.05)	0.154
DunedinPACE	0.00 (0.00, 0.01)	0.013
Epigenetic age acceleration at Y20		
IEAA	0.02 (-0.01, 0.06)	0.235
EEAA	0.11 (0.06, 0.15)	<.001†
PhenoAA	0.03 (-0.01, 0.08)	0.207
GrimAA	0.01 (-0.02, 0.03)	0.904
DunedinPACE	0.01 (0.00, 0.01)	0.018

Note: Point estimates (β) represent differences in EAA comparing participants per one-unit increase of ACE score (sum of each ACE item score) adverse childhood experiences; SES: socioeconomic status. † *P*-value less than 0.01 (Bonferroni correction threshold).

© 2023 Kim K et al. *JAMA Network Open.*

Supplementary Table 9. Associations between having any ACE (0 vs. 1+ ACEs) and epigenetic age acceleration (EAA) at Y15 and Y20

EAA estimators	Adjusted for demographics, early life SES, health-related behaviors and BMI, and adulthood SES	
	β (95% CI)	*P*-value
GEE models		
IEAA	0.01 (-0.90, 1.01)	0.914
EEAA	0.53 (-0.69, 1.77)	0.394
PhenoAA	-0.41 (-1.97, 1.14)	0.602
GrimAA	0.40 (-0.42, 1.23)	0.342
DunedinPACE	0.02 (-0.01, 0.05)	0.155
Epigenetic age acceleration at Y15		
IEAA	-0.11 (-0.61, 0.40)	0.688
EEAA	0.53 (-0.08, 1.16)	0.089
PhenoAA	-0.25 (-0.99, 0.48)	0.499
GrimAA	0.28 (-0.15, 0.73)	0.206
DunedinPACE	0.01 (0.00, 0.03)	0.015
Epigenetic age acceleration at Y20		
IEAA	-0.06 (-0.58, 0.45)	0.796
EEAA	0.59 (-0.01, 1.19)	0.051
PhenoAA	-0.66 (-1.39, 0.05)	0.071
GrimAA	0.14 (-0.31, 0.61)	0.532
DunedinPACE	0.01 (0.00, 0.03)	0.018

Note: Point estimates (β) represent differences in EAA comparing participants per one-unit increase of ACE score (sum of each ACE item score) adverse childhood experiences; SES: socioeconomic status. † *P*-value less than 0.01 (Bonferroni correction threshold).

© 2023 Kim K et al. *JAMA Network Open.*
