# Supplementary material for: Association of Adverse Childhood Experiences With Accelerated Epigenetic Aging in Midlife
Source: JAMA Netw Open. 2023 Jun 12;6(6):e2317987. doi: 10.1001/jamanetworkopen.2023.17987 (PMC10261996; doi:10.1001/jamanetworkopen.2023.17987)
Supplement: Supplement 2. — Data Sharing Statement [file jamanetwopen-e2317987-s002.pdf]

Data Sharing Statement

Kim. Association of Adverse Childhood Experiences With Accelerated Epigenetic Aging in Midlife. *JAMA Netw Open*. Published online June 12, 2023. doi:10.1001/jamanetworkopen.2023.17987

Data

Data available: No

Additional Information

Explanation for why data not available: CARDIA data are available upon reasonable request from the CARDIA Coordinating Center. CARDIA investigators are eager to collaborate with investigators interested in using CARDIA data. Please see the CARDIA website (https://www.cardia.dopm.uab.edu) for publications policies and for a list of CARDIA investigators. CARDIA data are also publicly available on the NIH-supported BioLINCC and dbGaP platforms.
